# Type IV Pilus Proteins Form an Integrated Structure Extending from the Cytoplasm to the Outer Membrane

**DOI:** 10.1371/journal.pone.0070144

**Published:** 2013-07-26

**Authors:** Chengyun Li, Regina A. Wallace, Wesley P. Black, Yue-zhong Li, Zhaomin Yang

**Affiliations:** 1 State Key Laboratory of Microbial Technology, School of Life Science, Shandong University, Jinan, China; 2 Department of Biological Sciences, Virginia Tech, Blacksburg, Virginia, United States of America; Loyola University Medical Center, United States of America

## Abstract

The bacterial type IV pilus (T4P) is the strongest biological motor known to date as its retraction can generate forces well over 100 pN. *Myxococcus xanthus*, a δ-proteobacterium, provides a good model for T4P investigations because its social (S) gliding motility is powered by T4P. In this study, the interactions among *M. xanthus* T4P proteins were investigated using genetics and the yeast two-hybrid (Y2H) system. Our genetic analysis suggests that there is an integrated T4P structure that crosses the inner membrane (IM), periplasm and the outer membrane (OM). Moreover, this structure exists in the absence of the pilus filament. A systematic Y2H survey provided evidence for direct interactions among IM and OM proteins exposed to the periplasm. For example, the IM lipoprotein PilP interacted with its cognate OM protein PilQ. In addition, interactions among T4P proteins from the thermophile *Thermus thermophilus* were investigated by Y2H. The results indicated similar protein-protein interactions in the T4P system of this non-proteobacterium despite significant sequence divergence between T4P proteins in *T. thermophilus* and *M. xanthus*. The observations here support the model of an integrated T4P structure in the absence of a pilus in diverse bacterial species.

## Introduction

*Myxococcus xanthus* is a gram-negative δ-proteobacterium that utilizes the type IV pilus (T4P) as a motility motor to move over solid surfaces [1]. Bacterial type IV pili (T4P), usually a few micrometers long and 6–7 nm in diameter, are polymeric protein filaments of the monomer pilin [2]–[4]. The T4P-mediated motility in *M. xanthus* is known as social (S) motility [2]. This is distinct from the adventurous (A) gliding motility of *M. xanthus* which is powered by an independent and different motility machinery [5]. T4P in rod-shaped bacteria such as *M. xanthus* are mostly localized to one of the two cell poles [2], [6], . Their retraction pulls a cell forward in *M. xanthus* S motility [8], [9] and in the twitching motility of the γ-proteobacterium *Pseudomonas aeruginosa*, the β-proteobacteria *Neisseria meningitidis* and *Neisseria gonorrhoeae* among other bacterial species [7], [10]. It is noteworthy that the T4P is the strongest among known biological motors as a single T4P can produce a stall force well over 100 pN when it retracts [8], [11]. *M. xanthus* S motility additionally requires extracellular polysaccharides (EPS) to function because *M. xanthus* EPS^−^ mutants are defective in S motility [12], [13]. The current model postulates that a T4P is triggered to retract at its cell proximal end when its distal end binds to EPS that are either associated with the *M. xanthus* cell surface or deposited on the gliding substratum [5].

About a dozen *pil* genes are required for T4P to function as a motor (see [1], [10], [14] and references therein). *pilA* encodes prepilin which is processed to mature pilin by a peptidase. *pilM*, *pilN*, *pilO*, *pilP* and *pilQ* usually form an operon in this gene order in *M. xanthus* and essentially all bacteria with these genes. PilM is a cytoplasmic protein that is likely anchored to the membrane by binding to the cytoplasmic tail of the bitopic transmembrane (TM) protein PilN. Like PilN, PilO is predicted to have a short cytoplasmic N-terminus, a TM helix and a periplasmic domain. PilP is an inner membrane (IM) lipoprotein exposed to the periplasm. PilQ is an outer membrane (OM) secretin which multimerizes to form a channel for the T4P to extend through the OM. PilC is predicted to be a polytopic TM protein with sizeable cytoplasmic domains. PilB and PilT are the two ATPases in the T4P system, the former responsible for T4P extension or assembly and the latter for retraction or disassembly.

In recent years, there have been various reports proposing a T4P IM complex consisting of PilM, PilN, PilO and PilP in *P. aeruginosa* and *Neisseria* [15]–[18]. Such a complex is consistent with the findings in the type II secretion system (T2SS) which is related to the T4P system evolutionarily [14], [19]. For example, the T2SS protein GspL has a single TM helix with a cytoplasmic and a periplasmic domain (GspL_cyto_ and GspL_peri_). GspL_cyto_ resembles PilM while GspL_peri_ is similar to PilN, providing evidence that PilM and PilN interactions are genuine. The lipoprotein PilP shares structural similarity with the TM protein GspC in the T2SS [20], [21]. Both PilQ and the T2SS protein GspD are members of the secretin family which form channels in the OM [14], [22]. How T4P proteins form a multicomponent machine for its motor function remains an active area of scientific inquiry.

This paper reports our investigation into the interactions among T4P proteins. Besides its motor function in S motility, T4P had been shown to regulate EPS production in *M. xanthus* [23]. More recently, a suppressor mutation in *pilB* was discovered that was capable of restoring EPS production to a *pilA* deletion mutant [24]. The analysis of genetic suppression here suggested an integrated T4P structure consisting of PilB, PilC, PilM, PilN, PilO, PilP and PilQ *in vivo*. The interactions among these proteins and the formation of this structure are likely independent of a T4P filament because these interactions were observed in a *pilA* deletion background. Using a yeast two-hybrid (Y2H) system, we demonstrate that the OM protein PilQ can be bridged inward to the IM through interactions in the periplasm with PilP. In addition, Y2H experiments also detected similar interactions among the T4P proteins of the non-proteobacterium *Thermus thermophilus*. Our findings support an integrated T4P structure capable of extending from the cytoplasm through the IM, periplasm and OM in bacteria on diverse branches of the phylogenetic tree. At least in the proteobacterium *M. xanthus*, such a structure may form in the absence of the pilus filament.

## Materials and Methods

### Strains and Growth Conditions

*M. xanthus* strains used in this study are listed in Table 1. They were grown using CYE medium [25] at 32°C. XL-1 Blue (Stratagene) was the *Escherichia coli* strain used for plasmid construction, which was grown using Luria-Bertani (LB) medium [26] at 37°C. All plates contained 1.5% agar except CYE soft agar plates which contained 0.4% agar. When necessary, kanamycin, oxytetracycline and ampicillin were supplemented at 100, 15 and 100 µg/ml, respectively, to CYE and/or LB for selection.

**Table 1:** *Myxococcus xanthus* strains used in this study.

Strains	Genotype	Reference
DK1622	Wild-type	[2]
DK8615	Δ*pilQ*	[34]
DK10410	Δ*pilA::tet*	[35]
DK10417	Δ*pilC*	This study
DK10416	Δ*pilB*	[60]
DK11122	Δ*pilI*	[33]
DK11133	Δ*pilH*	[33]
DK11135	Δ*pilG*	[33]
YZ690	Δ*pilA*	This study
YZ1181	Δ*pilH pilB^WA^*	This study
YZ1182	Δ*pilC pilB^WA^*	This study
YZ1183	Δ*pilG pilB^WA^*	This study
YZ1184	Δ*pilI pilB^WA^*	This study
YZ1185	Δ*pilM pilB^WA^*	This study
YZ1186	Δ*pilN pilB^WA^*	This study
YZ1187	Δ*pilO pilB^WA^*	This study
YZ1188	Δ*pilP pilB^WA^*	This study
YZ1189	Δ*pilQ pilB^WA^*	This study
YZ1190	Δ*pilA* Δ*pilQ pilB^WA^*	This study
YZ1191	Δ*pilA* Δ*pilQ*	This study
YZ1192	Δ*pilQ att::pilQ*	This study
YZ1573	Δ*pilA pilB^WA^*	This study
YZ1860	Δ*pilM*	This study
YZ1861	Δ*pilN*	This study
YZ1862	Δ*pilO*	This study
YZ1863	Δ*pilP*	This study
YZ2214	Δ*pilC att::pilC*	This study
YZ2215	Δ*pilN att::pilN*	This study
YZ2225	Δ*pilM att::pilM*	This study
YZ2226	Δ*pilO att::pilO*	This study
YZ2234	Δ*pilP att::pilP*	This study

Two *Saccharomyces cerevisiae* strains for the Y2H study, AH109 (*MATa*, *trp1-901*, *leu2-3*, *112*, *ura3-52*, *his3-200*, *gal4*Δ, *gal80*Δ, *LYS2::GAL1UAS-GAL1TATA-HIS3*, *GAL2UAS-GAL2TATA-ADE2*, *URA3::MEL1UAS-MEL1TATA-lacZ*) and Y187 (*MATα*, *ura3-52*, *his3-200*, *ade2-101*, *trp1-901*, *leu2-3*, *112*, *gal4*Δ, *met–*, *gal80*Δ, *URA3::GAL1UAS-GAL1TATA-lacZ*) (Clontech), were grown using YPDA medium [1% Yeast extract, 2% Peptone (Bacto), 2% glucose, 0.003% adenine hemisulfate (pH 6.5)] (Clontech). Synthetic dropout (SD) media with specified nutrients omitted were used for selection and phenotype analysis in Y2H experiments (see later). All yeast cells were grown at 30°C.

### Plasmids for *M. xanthus* Strain Construction

Two sets of plasmids (Table 2) were constructed for use in *M. xanthus*, one for deleting or replacing *M. xanthus* wild-type (WT) *pil* genes and the other for complementing *pil* deletions. All plasmids were confirmed by restriction digestions, polymerase chain reaction (PCR) and/or DNA sequencing.

**Table 2:** Plasmids used in this study.

Plasmids	Description	Reference
**Plasmids for use in ** ***M. xanthus***		
pBJ113	Vector, Kan^R^, *galK*	[28]
pCL153	*pilB^WA^* in pBJ113	This study
pCL179	*pilQ* in pWB425	This study
pMY7	Vector, Kan^R^, *galK*	Unpublished
pRW139	*pilN* in pWB425	This study
pRW141	*pilO* in pWB425	This study
pRW142	*pilM* in pWB425	This study
pRW143	*pilC* in pWB425	This study
pRW151	*pilP* in pWB425	This study
pWB425	Vector, Kan^R^, *att*	[31]
pWB600	Δ*pilM* in pMY7	This study
pWB601	Δ*pilN* in pMY7	This study
pWB602	Δ*pilO* in pMY7	This study
pWB603	Δ*pilP* in pMY7	This study
**Plasmids for use in Y2H experiment**		
pCL127	GAD-MxPilN	This study
pCL128	GAD-MxPilP	This study
pCL131	GAD-MxPilO	This study
pCL134	GBD-MxPilO	This study
pCL135	GBD-MxPilN	This study
pCL136	GBD-MxPilP	This study
pCL137	GAD-MxPilM	This study
pCL138	GBD-MxPilM	This study
pCL141	GAD-MxPilQc	This study
pCL142	GBD-MxPilQc	This study
pCL150	GBD-MxPilQ′	This study
pCL152	GAD-MxPilQ′	This study
pCL180	GAD-TtPilN	This study
pCL181	GBD-TtPilN	This study
pCL182	GAD-TtPilO	This study
pCL183	GBD-TtPilO	This study
pCL184	GAD-TtPilW	This study
pCL185	GBD-TtPilW	This study
pCL186	GAD-TtPilQ_0_	This study
pCL187	GBD-TtPilQ_0_	This study
pCL188	GAD-TtPilQ_01_	This study
pCL189	GBD-TtPilQ_01_	This study
pCL192	GAD-TtPilQ_012_	This study
pCL193	GBD-TtPilQ_012_	This study
pGAD	pGADT7, Amp^R^, Leu	Clontech
pGAD-T	Amp^R^, Leu	Clontech
pGBD	pGBKT7, Kan^R^, Trp	Clontech
pGBD-53	Kan^R^, Trp	Clontech

The plasmids for *pil* gene deletions were pWB600 (Δ*pilM*), pWB601 (Δ*pilN*), pWB602 (Δ*pilO*) and, pWB603 (Δ*pilP*) [27]–[29]. These plasmids were constructed using pMY7 which is pZErO (Invitrogen) containing the *Aeromonas hydrophila galK* gene [30]. Deletion alleles, which were obtained using a two-step PCR procedure as described previously [31], were cloned into pMY7 digested with *Bam*HI and *Eco*RI. The Δ*pilM* allele deleted codons from 8 to 392, Δ*pilN* from 6 to 222, Δ*pilO* from 8 to 189 and Δ*pilP* from 7 to 197 of their respective genes. In addition, pCL153 (*pilB^K327A^* or *pilB^WA^*) was used for the replacement of WT *pilB* (*pilB^+^*) with *pilB^WA^* (WA stands for Walker A), which was obtained from pWB630 [24] by digestion with *Hin*dIII and *Xba*I and cloned into the same sites in pBJ113.

The plasmids for complementation were pRW143 (*pilC*), pRW142 (*pilM*), pRW139 (*pilN*), pRW141 (*pilO*), pRW151 (*pilP*), and pCL179 (*pilQ*). These plasmids were constructed using the *M. xanthus* expression plasmid pWB425 as the vector [31]. The target genes were PCR amplified using primers containing *Kpn*I and *Bam*HI at the 5′ and 3′, respectively. These fragments were cloned into the same restriction sites in pWB425. Relative to the coding regions of each gene, pRW143 contains a fragment from 19 base pairs (bp) upstream to 13 bp downstream of *pilC*, pRW142 from 17 bp upstream to 15 bp downstream of *pilM*, pRW139 from 14 bp upstream to 17 bp downstream of *pilN*, pRW141 from 60 bp upstream to 20 bp downstream of *pilO*, pRW151 from 79 bp upstream to 49 pb downstream of (*pilP*), and pCL179 from 18 bp upstream to 25 bp downstream of (*pilQ*).

### *M. xanthus* Strain Construction

All *M. xanthus* strains (Table 1) are isogenic to the laboratory WT strain DK1622 [2]. The plasmids pWB600 through pWB603 (Table 2) with deletion alleles of *pilM* through *pilP* were used for the construction of deletion mutants using DK1622 as the parent as described previously [31]. The resultant single *pil* deletion mutants are YZ1860 (Δ*pilM*), YZ1861 (Δ*pilN*), YZ1862 (Δ*pilO*), and YZ1863 (Δ*pilP*). pCL153 were then used to replace *pilB^+^* with *pilB^WA^* in these strains to construct YZ1185 through YZ1188. In addition, pCL153 was used to replace *pilB^+^* in DK10417 (Δ*pilC*), DK11135 (Δ*pilG*), DK11133 (Δ*pilH*), DK11122 (Δ*pilI*) and DK8615 (Δ*pilQ*) to construct YZ1182, YZ1183, YZ1181, YZ1184 and YZ1189, respectively. To construct YZ1190 and YZ1191, genomic DNA of DK10407 (Δ*pilA::tet*) was transformed into YZ1189 (Δ*pilQ pilB^WA^*) and DK8615 (Δ*pilQ*) [32]–[35].

For the complementation of single deletions of *pilC*, *pilM*, *pilN*, *pilO*, *pilP* and *pilQ*, the pRW series of plasmids (Table 2) were transformed into their corresponding deletion strains to construct YZ2214, YZ2225, YZ2215, YZ2226, YZ2234 and YZ1192, respectively.

### Assays for S motility and EPS Production

*M. xanthus* cells in exponential growth were harvested and resuspended in MOPS (morpholinepropanesulfonic acid) buffer (10 mM MOPS (pH 7.6), 2 mM MgSO_4_) at 5.0×10^9^ cells/ml. For the S-motility assay, 5 µl of the cell suspension was spotted to the center of a soft agar plate which was documented after 5 days of incubation. For EPS analysis, 5 µl of the cell suspension was placed on a CYE plate supplemented with Calcofluor white at 50 µg/ml. Plates were incubated for 6 days and the florescence was documented under ultraviolet (UV) illumination at ∼365 nm [23], [29], [31].

### Plasmids for Y2H Experiments (Table 2)

The MATCHMAKER System 3 from Clontech was used for the Y2H experiments in this study. The two cloning vectors pGAD (pGADT7) and pGBD (pGBKT7) allow proteins to be fused to the C-terminus of the GAL4 transcription activation domain (GAD) and GAL4 DNA binding domain (GBD), respectively [36]. A fragment with the coding region of interest of a gene from either *M. xanthus* or *T. thermophilus* was amplified by PCR and cloned into both pGAD and pGBD restricted by appropriate endonucleases. pGAD-T and pGBD-53, which contain fusions to T-antigen and p53, are provided by Clontech as positive controls.

pCL127, pCL128 and pCL131contain *M. xanthus* (Mx) PilN, PilP and PilO fused to GAD whereas pCL135, pCL136 and pCL134 contain the same proteins fused to GBD, respectively. Note that the signal peptide of PilP and the TM helices of PilN and PilO are excluded from these constructs. pCL150 and pCL152 contain the same MxPilQ fragment truncated at the C-terminus (PilQ′) in pGBD and pGAD; pCL141 and pCL142 contain the Secretin_N region at the center of MxPilQ (PilQc) in the two Y2H vectors.

Plasmids pCL180 through pCL193 contain fusions of *T. thermophilus* (Tt) Pil proteins to GAL4 in Y2H vectors (Table 2). pCL180, pCL182 and pCL184 contain TtPilN, TtPilO and TtPilP fused to GAD and pCL182, pCL183 and pCL185 contain the same protein fragments fused to GBD, respectively. The TtPilQ N-terminus is divided into N0, N1 and N2 subdomains. pCL186 through pCL193 contain N0 (PilQ_0_), N0 and N1 (PilQ_01_) and all three subdomains (PilQ_012_) in pGAD and pGBD, respectively.

### Y2H Mating Protocol

The plates used for the Y2H mating protocol are SD without (−) tryptophan (Trp), leucine (Leu), histidine (His), adenine (Ade) or their combinations (Clontech). The mating protocol in the manual for the MATCHMAKER system (Clontech) was followed to examine systematic protein-protein interactions in Y2H. Briefly, a pGAD-derived plasmid was transformed into Y187 by selection on SD-Leu and a pGBD-derived plasmid into AH109 by selection on SD-Trp plates. 20 µl of culture of each transformant was placed in the same well of a 96-well plate seeded with 160 µl of YPDA each. After incubation for 18 hours on a rotary shaker, 5 µl of the culture from each well was transferred onto a 150×15 mm^2^ petri dish with SD-Trp-Leu medium in the same 96-well plate format. After 48–72 hours of incubation, cells were replica plated to SD-Ade and SD-His plates. After 3–5 days of incubation, growth on these plates was scored for the His^+^ or His^−^ and Ade^+^ or Ade^−^ phenotypes. Note that both SD-His and SD-Ade plates were without Trp and Leu. The SD-His plates were additionally supplemented with 2.5 mM 3-Amino-1, 2, 4-triazole (3-AT) which is a competitive inhibitor of the His3 enzyme.

### Y2H by Co-transformation

A pair of pGAD- and pGBD-derived plasmids were co-transformed [37] into the yeast strain AH109 by selection on SD-Leu-Trp plates. Transformants were then examined for growth on SD-Ade and SD-His plates and for the expression of β-galactosidase. For the analysis of growth on SD-Ade and SD-His plates, cells in exponential growth were harvested and resuspended in SD medium. 5 µl of cell suspensions at 4×10^6^, 8×10^5^, 1.6×10^5^ and 3.2×10^4^ cells per ml were placed on SD-Ade and SD-His plates in a row from left to right. The growth was documented by photographs after 3 days of incubation. The analysis of β-galactosidase activity was performed as described by the Yeast Protocols Handbook (Clontech) using ONPG (o-nitrophenyl β-D-galactopyranoside) as the substrate. 1 unit of β-galactosidase is defined as the amount that hydrolyzes 1 µmol of ONPG per minute per cell.

## Results and Discussion

### Deletions of *M. xanthus pilM*, *pilN*, *pilO*, *pilP, pilQ* and *pil*C Can Be Complemented

**Figure 1:**
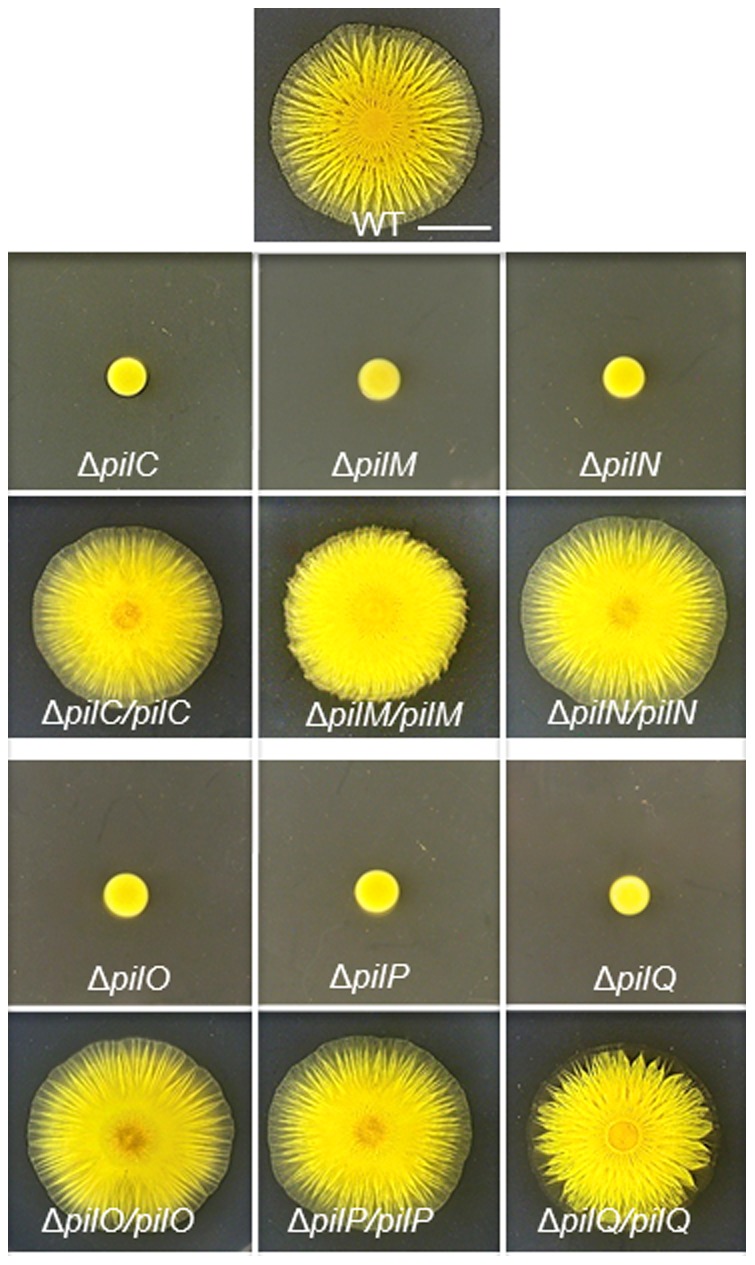
*pil* deletions can be complemented *in trans*. S motility was analyzed by colony spreading (or swarming) on soft agar plates as described in Materials and Methods. The *pil* deletion strains are DK10417 (Δ*pilC*), YZ1860 (Δ*pilM*), YZ1861 (Δ*pilN*), YZ1862 (Δ*pilO*), YZ1863 (Δ*pilP*) and DK8615 (Δ*pilQ*). The complemented stains are YZ2214 (Δ*pilC/pilC*), YZ2225 (Δ*pilM/pilM*), YZ2215 (Δ*pilN/pilN*), YZ2226 (Δ*pilO/pilO*), YZ2234 (Δ*pilP/pilP*) and YZ1192 (Δ*pilQ/pilQ*). The WT strain DK1622 was included on the top center as a control and the scale bar represents 5 mm. See Table 1 for more precise genotypes of the strains.

It was observed previously that individual *P. aeruginosa pilM*, *pilN*, *pilO* and *pilP* mutants could not be complemented by their respective WT genes *in trans* unless other genes in the same gene cluster or operon were provided as well [15]. These observations were taken as part of the evidence to conclude that PilM, PilN, PilO and PilP formed an inner membrane (IM) complex critical for the stability of the PilQ secretin on the outer membrane (OM) [15]. A similar approach was taken here to examine the complementation of *M. xanthus pil* mutations as an attempt to study interactions among *M. xanthus* T4P proteins. Because *pilM*, *pilN*, *pilO*, *pilP* and *pilQ* are likely in an operon in *M. xanthus* as in most other bacteria [1], [10], in-frame deletions of these *M. xanthus* genes were constructed to minimize polar effects (see Materials and Methods). A *pilC* deletion was also constructed because PilC may play a key role in organizing an IM complex since it is the only predicted polytopic TM T4P protein [10]. When examined on soft agar plates (Figure 1), all mutants were found to be defective in S motility as anticipated since none was expected to assemble T4P [1], [38].

Next, plasmids were constructed for complementation of these deletion mutants. Six fragments containing *pilC*, *pilM*, *pilN*, *pilO*, *pilP* and *pilQ*, respectively, were cloned into an expression vector [31] which is able to integrate at the Mx8 phage attachment site (*att*) on the *M. xanthus* chromosome. The resulting plasmids were transformed into their respective deletion strains and the transformants were examined on soft agar plates for S motility (Figure 1) which requires fully functional T4P. Unlike the observations in *P. aeruginosa* [15], these transformants all showed S motility similar to the WT. It is noted that expression of *pil* genes *in trans* in these complemented strains are likely lower than in the WT *in situ* [24], [39]. The previous observation [15] could be explained if T4P assembly is more sensitive to an imbalance of proteins forming a complex in *P. aeruginosa* than in *M. xanthus*. Alternatively, the small insertions or scar mutations in *P. aeruginosa pil* genes [15] could be partially polar on downstream genes.

### *pilB^WA^* Suppresses the EPS Defect of Δ*pilG*, Δ*pilH* & Δ*pilI*, But Not That of Δ*pilC*, Δ*pilM*, Δ*pilN*, Δ*pilO*, Δ*pilP*, or Δ*pilQ*

**Figure 2:**
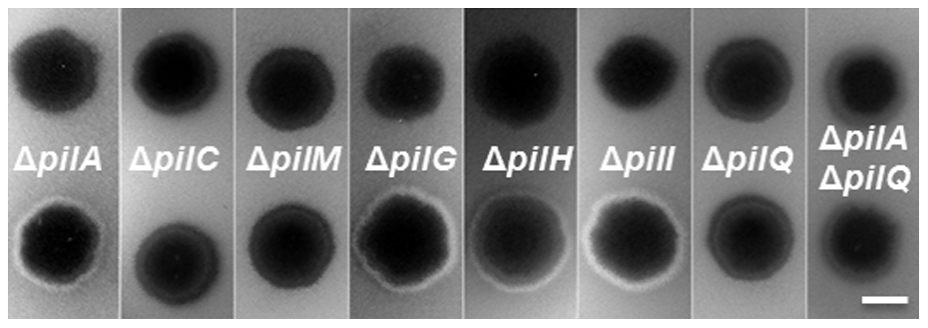
*pilB^WA^* suppresses some but not all T4P^−^ *pil* deletions in EPS production. EPS levels were analyzed on plates with the fluorescent dye Calcofluor white (See Materials and Methods). The presence of fluorescence indicates EPS^+^ and its absence EPS^−^. Each strip shows two strains, the upper is a strain with the indicated mutation(s) in a *pilB^+^* background and the lower in the *pilB^WA^* background. Strains used are YZ690 (Δ*pilA*), YZ1573 (Δ*pilA pilB^WA^*), DK10417 (Δ*pilC*), YZ1182 (Δ*pilC pilB^WA^*), YZ1860 (Δ*pilM*), YZ1185 (Δ*pilM pilB^WA^*), DK11135 (Δ*pilG*), YZ1183 (Δ*pilG pilB^WA^*), DK11133 (Δ*pilH*), YZ1181 (Δ*pilH pilB^WA^*), DK11132 (Δ*pilI*), YZ1184 (Δ*pilI pilB^WA^*), YZ1191 (Δ*pilA* Δ*pilQ*) and YZ1190 (Δ*pilA* Δ*pilQ pilB^WA^*). *pilB^WA^* failed to suppress Δ*pilN*, Δ*pilO*, Δ*pilP* and Δ*pilQ* as it did Δ*pilC* and Δ*pilM* (data not shown).

Taking advantage of a newly constructed gain-of-function mutation in *pilB*, an alternative genetic approach was explored to examine if T4P proteins form an integrated structure in *M. xanthus*. PilB, a cytoplasmic ATPase in the T2SS ATPase superfamily, has been shown to function as the T4P assembly ATPase with Δ*pilB* mutations leading to a T4P^−^ phenotype [6], [40]. Previous results indicated that Δ*pilA* or any other *pil* mutants that were T4P^−^ were also defective in EPS production, indicating a role for T4P in EPS regulation in *M. xanthus* [23]. Recently a *pilB* mutation was found that restored EPS production to a Δ*pilA* mutant [24]. This mutation, which is referred to as *pilB^WA^* or *pilB^K327A^*, resulted in the substitution of the strictly conserved lysine (K) 327 with an alanine (A) in the signature Walker A box of such ATPases. To examine if *pilB^WA^* could suppress the EPS^−^ phenotype resulting from other T4P^−^ mutations, the *pilB^+^* allele was replaced by *pilB^WA^* in the deletion mutants of *pilC*, *pilM*, *pilN*, *pilO*, *pilP*, and *pilQ* as well as *pilG*, *pilH*, and *pilI*, respectively. The resulting strains were examined on plates containing Calcofluor white, a fluorescent dye that binds to *M. xanthus* EPS (Figure 2). As indicated by fluorescence, *pilB^WA^* suppressed Δ*pilG*, Δ*pilH* and Δ*pilI* in EPS production, but not Δ*pilC*, Δ*pilM*, Δ*pilN*, Δ*pilO*, Δ*pilP*, or Δ*pilQ*.

The lack of suppression of Δ*pilQ* by *pilB^WA^* was further investigated. PilQ is the secretin that forms a multimeric channel in the OM to allow the passage of the pilus filament through the OM [38]. It was previously reported that a *N. meningitidis pilQ* mutant assembled T4P in the periplasm likely because a *pilQ* mutant in an otherwise WT background can still assemble the pilus filament in the periplasm even though it fails to extend through the OM [41], [42]. In addition, mutant PilA or pilins trapped in the IM may negatively influence EPS production [43]. In a *pilQ* mutant, either periplasmic pili and/or an increase in unassembled pilins in the IM could down regulate EPS levels. To examine if the lack of suppression of Δ*pilQ* by *pilB^WA^* was due to these reasons, we constructed a Δ*pilA* Δ*pilQ pilB^WA^* triple mutant. As shown in Figure 2, *pilB^WA^* still failed to restore EPS production to a Δ*pilQ* Δ*pilA* mutant. Because *pilB^WA^* suppresses Δ*pilA* (Figure 2) [24], the results here indicate that PilB^WA^ requires PilQ to signal EPS production in *M. xanthus* in a Δ*pilA* background or the absence of a pilus.

What are the explanations for this pattern of suppression of *pil* mutations by *pilB^WA^*? The suppression of Δ*pilG*, Δ*pilH* and Δ*pilI* was perhaps not surprising. PilG, PilH and PilI are essential for the translocation of pillin across the IM [33] and their mutants in essence may resemble a Δ*pilA* mutant due to the lack of pilins and pili. The lack of suppression of Δ*pilC* and Δ*pilM* may also have simple explanations. PilM is a cytoplasmic protein [44] and the multi-TM protein PilC is predicted to have sizeable cytoplasmic loops [45]. A frugal explanation would be that PilB^WA^ requires interactions with both PilC and PilM directly or through each other in the cytoplasm in order to signal EPS production. The suppression of Δ*pilG*, Δ*pilH* and Δ*pilI* by *pilB^WA^* as well as the lack of suppression of Δ*pilC* and Δ*pilM* may therefore be explained parsimoniously with little difficulty.

Without invoking multiple direct and indirect interactions, however, there would be no good explanation for the lack of suppression of Δ*pilN*, Δ*pilO*, Δ*pilP* and Δ*pilQ* by *pilB^WA^*. As has been mentioned in Introduction, PilQ forms an oligomeric passage for the T4P filament on the OM. PilP is a lipoprotein anchored to the IM by a lipid moiety [46]. PilO and PilN are both predicted to have similar membrane topology, each with a cytoplasmic N-terminal tail of a dozen residues, a TM helix followed by the C-terminus in the periplasm [47], [48]. It is known that the N-terminal tail of PilN interacts with PilM in the cytoplasm [44]. Taking into consideration the above structural information and the suppression patterns of various *pil* mutations by *pilB^WA^* in *M. xanthus*, a reasonable explanation assumes a multi-component complex consisting of all these proteins. To explain the requirement of PilQ for EPS production in a *pilB^WA^* background, for example, PilQ in the OM must be connected to the cytoplasmic PilM and/or the IM spanning PilC to affect the activity of PilB^WA^. This connection through the periplasm and the IM may involve PilP, PilO and/or PilN, but not necessarily the pilus filament. Such a model would explain how PilQ could influence the activity of PilB^WA^ in the cytoplasm and why *pilB^WA^* suppresses none of the deletions of *pilC*, *pilM*, *pilN*, *pilO* and *pilP*. That is, PilB^WA^, which likely represents a particular conformation of PilB *in vivo* [24], must communicate either directly or indirectly with the IM protein PilC and the cytoplasmic protein PilM anchored to the IM by PilN [45]. The other proteins, PilO, PilP and PilQ, must in turn affect the activity of PilB^WA^ indirectly through their interactions with PilC, PilM and/or PilN.

### Interactions Among *M. xanthus* PilN, PilO, PilP and PilQ in Y2H System

The above model envisions an integrated T4P structure with extensive protein-protein interactions that were investigated more directly using the Y2H system. The Y2H mating protocol (See Materials and Methods) was utilized here since it can be used to examine interactions among large numbers of proteins and their domains. In this protocol, a protein or its domain is fused to the C-termini of both the GAL4 activation domain (GAD) and DNA binding domain (GBD) in pGAD and pGBD vectors, respectively (Table 2). The two fusion plasmids are then transformed into two yeast reporter strains of the opposite mating types (Y187: *MATα* and AH109: *MATa*), respectively. The two strains are then mated with each other and with others expressing fusions to a different GAL4 domain. After mating, the diploid cells containing a pGAD- and a pGBD-derived plasmids are examined on selective plates without histidine (SD-His) or adenine (SC-Ade) for His^+^ and Ade^+^ phenotypes as indicators of the expression of two reporter genes of the Y2H system.

**Figure 3:**
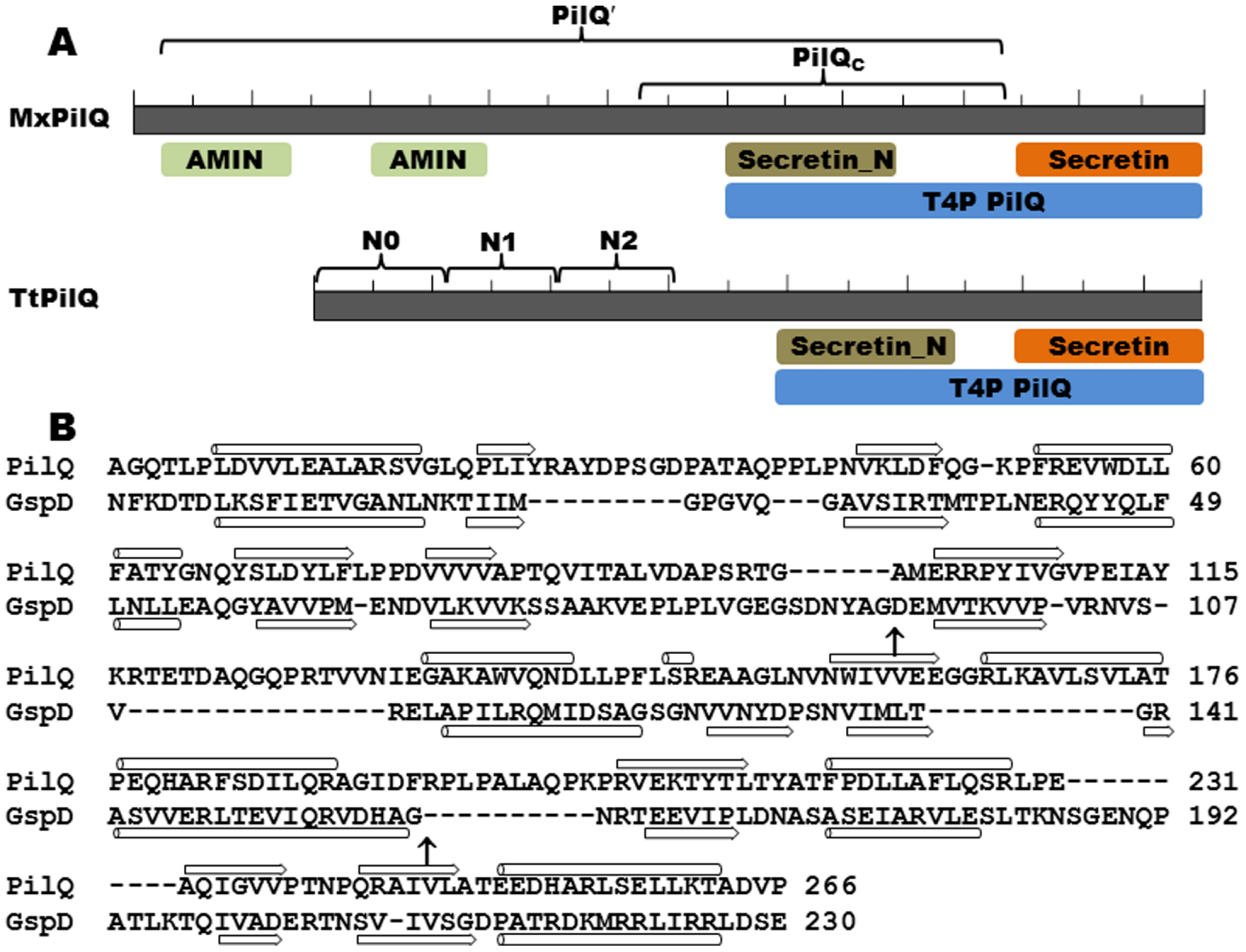
Conserved domains of PilQ. Panel **A.** Conserved domains of MxPilQ (901 residues) and TtPilQ (757 residues) [58]. The conserved feature are drawn to scale and areindicated by shaded letters below both protein. Each has the PilQ region at their C-terminus consisting of the highly conserved Secretin domain and the region immediately N-terminal of secretin (Secretin_N). Two AMIN domains [59] are found at the N-terminus of MxPilQ but not TtPilQ. The brackets and the labels above indicate the different Y2H constructs and/or subdomains in each protein. Panel **B.** Structural alignment of the N-termini of TtPilQ and the T2SS secretin GspD from Enterotoxigenic *E. coli* (ETEC) [50], [55]. The boundaries between N0 and N1 as well as N1 and N2 subdomains in GspD are indicated by arrows (↑). The secondary structure of GspD from crystallography and that of TtPilQ predicted from modeling are indicated below and above the aligned sequences, respectively, with β strands represented by block arrows and α helices by cylinders.

Table 3 represents results for PilN, PilO, PilP and PilQ using this mating protocol. The constructs for PilN and PilO excluded their N-terminal tails and TM domains and those for PilP excluded its predicted lipoprotein signal peptide at the N-terminus [49]. PilQ constructs were designed based on primary sequence conservation (Figure 3A) and secondary structure predictions [50], [51] (data not shown). The PilQ′ construct (Figure 3A and Table 1) excluded the C-terminal secretin region which is predicted to form β-barrels in the OM membrane [51]; the PilQc construct contained the conserved Secretin_N region immediately N-terminal of the more highly conserved secretin domain (Figure 3A). In this experiment, seven pairs of plasmids conferred growth on selective media (Table 3). The results suggested the following four pairwise interactions: PilN-PilO, PilO-PilP, PilP-PilQ and PilQ-PilQ. PilP interacted with both PilQ′ and PilQc (Figure 3A) but not with two other constructs containing the N-termini of PilQ truncated before Secretin_N (data not shown). Therefore, the Secretin_N region may solely contribute to the interaction of PilQ with PilP in *M. xanthus*. PilP and PilQ′ were the only pair whose interactions were detected in both orientations in reciprocal Y2H vectors while three of the GAL4 fusions (GAD-PilN, GBD-PilO and GBD-PilQc) gave no positive interactions in this Y2H experiment (Table 3). It is noteworthy that the four pairs of interactions detected here (Table 3) are all predicted to be in the periplasm of *M. xanthus*. Various fragments of PilB, PilM and PilC were also examined, but no interaction between them was detected using this Y2H mating protocol (data not shown).

**Table 3:** Interactions among *M. xanthus* Pil proteins detected by Y2H mating protocol.

*	PilN	PilO	PilP	PilQ′	PilQ_C_	p53
PilN	−	−	−	−	−	−
PilO	+	−	+	−	−	−
PilP	−	−	−	+	−	−
PilQ′	−	−	+	+	−	−
PilQ_C_	−	−	+	+	−	−
T-antigen	−	−	−	−	−	+

* The first column and first row indicate proteins fused to GAD and GBD, respectively. Diploid cells containing the indicated pair of fusions were examined for growth on SD-His and SD-Ade plates. Plus (+), growth on both plates; minus (−), growth on neither plates.

**Figure 4:**
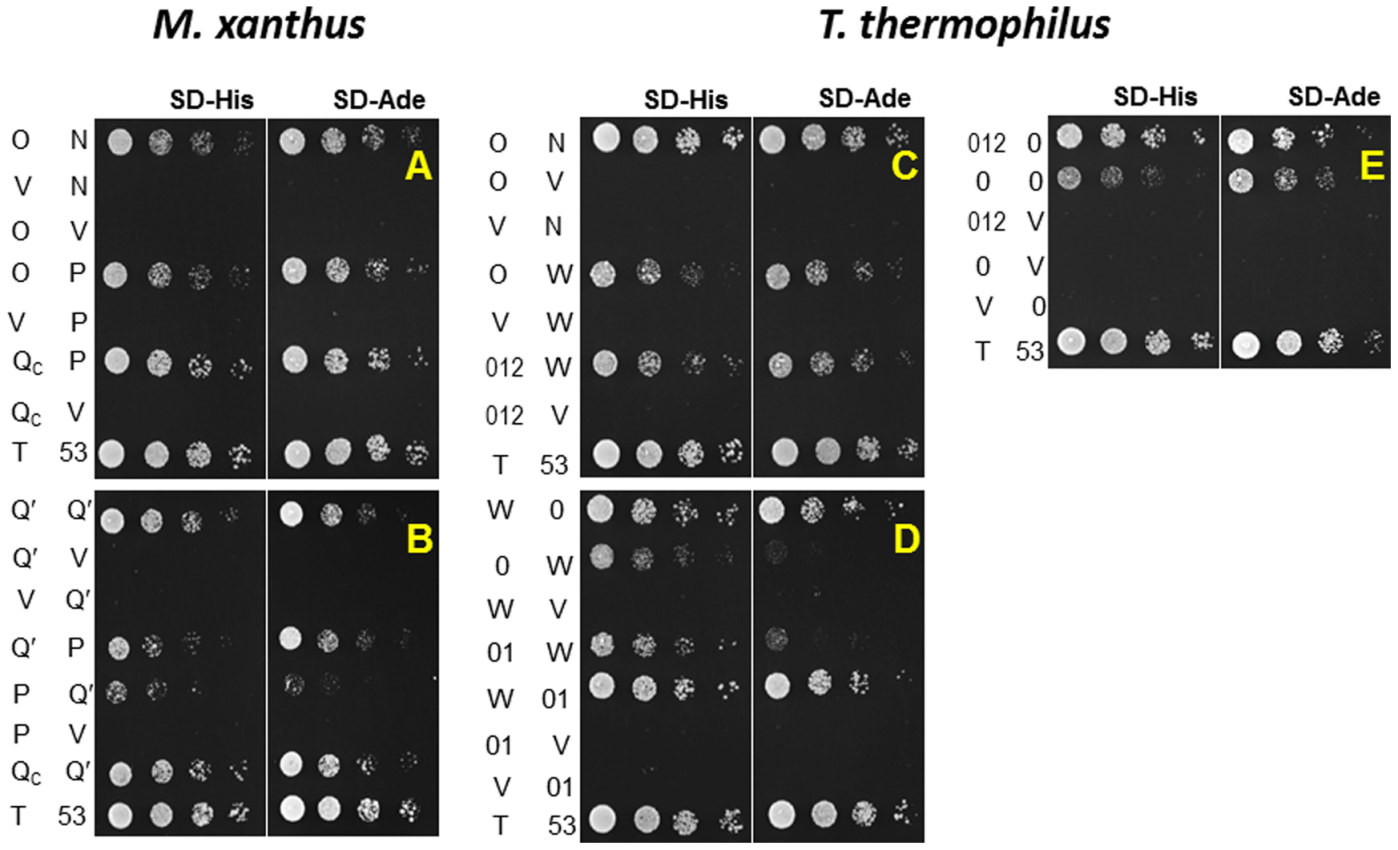
Pairwise interactions among Pil proteins in Y2H system. Panels **A** and **B**. Interactions among *M. xanthus* Pil proteins. Panels **C, D** and **E**. Interactions among *T. thermophilus* Pil proteins. The first and second columns on the left of each panel indicate Pil proteins or fragments fused to GAD and GBD in Y2H plasmids by their last letter, respectively. V indicates an empty Y2H vector. N0, N1, N2 and their combinations are represented by their numerals only. Last row in each panel contained the positive control with T-antigen (T) and p53 (53). The left half of each panel shows growth on SD-His and the right on SD-Ade plates, respectively. The spots in each row in a panel were inoculated by serial dilutions of the same yeast cells with the indicated Y2H plasmids. See text and Materials and Methods for details.

**Figure 5:**
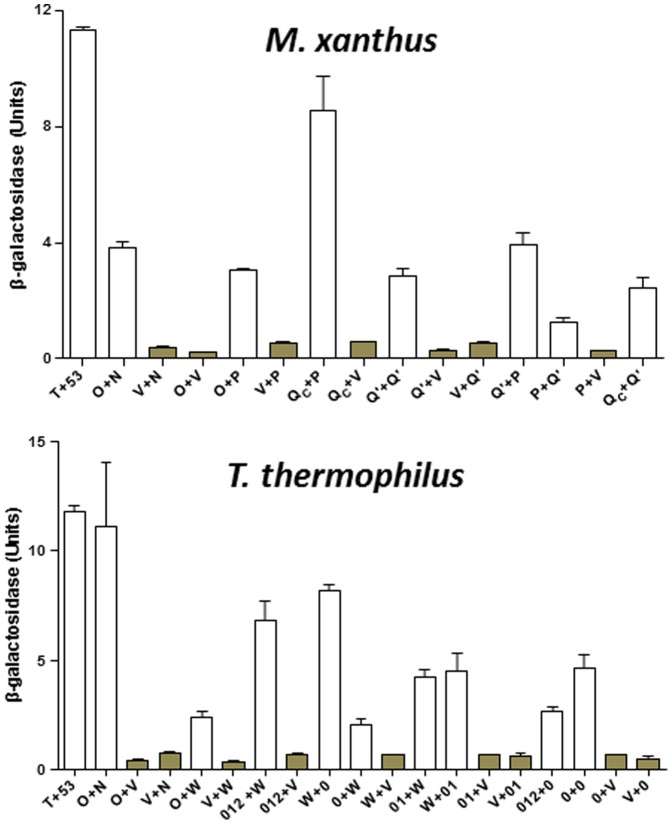
Quantification of β-galactosidase in Y2H experiment. The upper and lower panels show the β-galactosidase activity for Pil protein interactions in Y2H experiments from *M. xanthus* and *T. thermophilus*, respectively. The values for β-galactosidase activity were the average of three independent experiments and samples in each experiment were analyzed in triplicate. See Figure 4 for protein designations under each panel. The bars for the vector controls are shaded for comparison. See text and Materials and Methods for more details.

The interactions detected by the mating protocol were verified by co-transformation of the yeast strain AH109 and additional analysis of the expression of reporter genes. Briefly, each of the seven pairs of plasmids that gave positive interactions (Table 3) was co-transformed into AH109 and the transformants were examined phenotypically. As a semi-quantitative analysis of the strength of interactions, 5-fold serial dilutions of the transformants were spotted on SD-His and SD-Ade plates as shown in Figures 4A and 4B. The pairs that showed positive interactions in the mating protocol (Table 2) all showed growth on these two selective plates. The expression of β-galactosidase, the third reporter of the Y2H system, confirmed these positive interactions (Figure 5A). When each of the plasmids was co-transformed with an empty Y2H vector, none of them led to the expression of the three reporters (Figures 4A, 4B & 5A), eliminating the possibility of autoactivation by any single GAL4 fusion.

In summary, the PilQ-PilQ interaction is consistent with its oligomerization in the OM. The PilQ-PilP, PilP-PilO and PilO-PilN pairwise interactions connect PilQ to PilN which interacts with PilM in the cytoplasm [44]. These results are therefore supportive of a model wherein the OM secretin is connected to the cytoplasmic ATPase PilB through a series of physical interactions as proposed based on the suppression of *pil* mutations by *pilB^WA^* in EPS production (Figure 2). The pilus filament is apparently not required for the formation of this integrated T4P complex as *pilB^WA^* suppresses Δ*pilA* yet not a Δ*pilA* Δ*pilQ* double mutation (Figure 2).

### Similar Interactions Occur Among *T. thermophilus* T4P Proteins

T4P systems are found in diverse lineages in bacteria [7], [10], but the best experimental systems for T4P studies, which include *M. xanthus*, *P. aeruginosa*, and *Neisseria* sp., are proteobacteria [10]. We used the Y2H system to investigate whether the interactions reported here for *M. xanthus* are conserved in the non-proteobacterium *T. thermophilus* (Tt), a thermophile in the deep-rooted Deinococci-Thermus phylum [52]. The motivation for this choice included the prevalent use of thermophilic proteins for structural studies including *T. thermophilus* T4P proteins [44], [45], [53]. The conservation of protein-protein interactions, if true, would allow the insights from structural studies of *T. thermophilus* T4P proteins to be applied to experimentally more accessible systems, and vice versa.

Most T4P proteins and their domains are well conserved in different organisms and so is the gene order in the *pilM*, *pilN*, *pilO*, *pilP* and *pilQ* gene cluster [10]. There are exceptions when it comes to PilQ and PilP, however [54]. As shown in Figure 3A, except the highly conserved secretin domain and the adjacent Secretin_N region, there is little homology between *T. thermophilus* PilQ (TtPilQ) and *M. xanthus* PilQ (MxPilQ) with the latter over 140 residues longer. Moreover, instead of the IM lipoprotein PilP, the gene between *pilO* and *pilQ* in *T. thermophilus* encodes PilW (TtPilW), which is predicted to have a single TM helix with a periplasmic region without homology to any known T4P or T2SS proteins from other bacterial lineages [54]. However, because *pilW* is in the same chromosomal location as *pilP* relative to other *pil* genes, it is possible that PilW may have similar functions as PilP in bridging PilQ to the IM and to other IM T4P proteins in *T. thermophilus*. The interactions among *T. thermophilus* PilN, PilO, PilW and PilQ were therefore investigated by Y2H for comparison with *M. xanthus* T4P proteins.

We first examined the interactions among *T. thermophilus* PilW, PilO and PilN by cloning their C-termini truncated immediately after their predicted TM helices into the Y2H vectors (Table 2). Pairs of pGAD- and pGBD-derived fusion plasmids were then transformed into the Y2H reporter strain AH109. As shown in Figure 4C, transformants of PilO and PilW constructs as well as those of PilO and PilN grew on SD-His and SD-Ade plates. These observations were further validated by the expression of β-galactosidase (Figure 5B). The results indicate that despite sequence divergence, PilW as well as PilO and PilN interact similarly in *T. thermophilus* as PilP, PilO and PilN in *M. xanthus*.

Next, we examined the interactions between TtPilW and TtPilQ. Recall that PilP interacted with the Secretin_N region of MxPilQ in Y2H (PilQc construct in Figures 3A and 4A). Therefore, we tested the interaction of TtPilW with the Secretin_N region of TtPilQ (Figure 3A) using Y2H, but the relevant yeast co-transformants grew on neither SD-His nor SD-Ade (data not shown). Interestingly, while the N-terminus of TtPilQ shares no homology with *M. xanthus* PilQ (Figure 3A), it does have limited similarity with the periplasmic N-terminus of the T2SS secretin GspD (GspD_peri_) (Figure 3B). The structure of the N-terminus of TtPilQ can in fact be modeled using the structure of GspD_peri_ as a template [50], [55]. The secondary structure prediction of this modeling is shown in Figure 3B. Using the structures of GspD_peri_ [55] as a guide, the N-terminus of TtPilQ_peri_ can be similarly divided into N0, N1 and N2 subdomains (Figure 3B). While these GspD_peri_ subdomains resemble one another in structure to some degree [55], only N0 was shown to interact with GspC which is an ortholog of PilP in T2SS [20]. TtPilW showed interactions with N0 and the combination of N0 and N1 of TtPilQ (TtPilQ_0_ and TtPilQ_01_) in Y2H in both orientations as indicated by growth on SD-His and SD-Ade plates (Figure 4D) and by expression of β-galactosidase (Figure 5B). TtPilW was also detected to interact with the three subdomains combined (TtPilQ_012_) in one orientation (Figure 4C) but not with N1 or N2 individually (data not shown). The strengths of interactions of TtPilW with TtPilQ_0_ and with TtPilQ_01_ in both orientations were similar as indicated by growth and β-galactosidase activity (Figures 4D & 5B). These observations suggest that like GspC, TtPilW interacts with the N0 subdomain of its cognate secretin despite the lack of any detectable similarity between TtPilW with GspC or PilP at the level of their primary [54] or predicted higher order structures (data not shown). Such interactions allow the bridging of PilQ to the IM in both *T. thermophilus* and *M. xanthus*.

The interaction of TtPilQ with itself was also investigated. Constructs similar to *M. xanthus* PilQ′ and PilQc gave no indication of interaction using Y2H (data not shown). We additionally examined constructs containing N0, N1 and N2 in various combinations. Only two pairs among them were found to confer interactions in Y2H as shown in Figures 4E and 5B; these are TtPilQ_0_ with itself and with TtPilQ_012_. It is interesting to note that in the Y2H system, *M. xanthus* PilQ-PilQ interactions are mediated by Secretin_N whereas *T. thermophilus* PilQ-PilQ interactions by N0 (Figures 3, 4 and 5). Nevertheless, these results are consistent with the conservation of structural interactions in the periplasm and the multimerization of PilQ in both the proteobacterium *M. xanthus* and the non-proteobacterium *T. thermophilus*.

## Concluding Remarks

**Figure 6:**
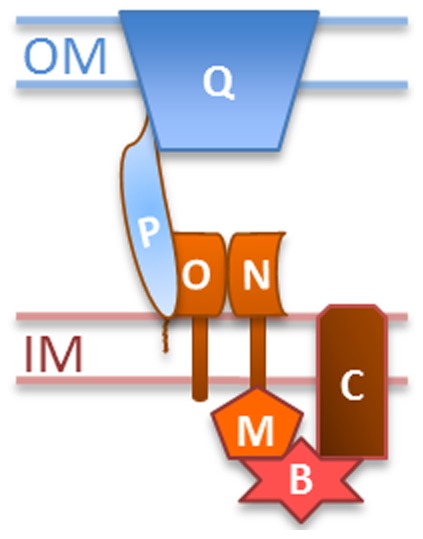
An integrated T4P structure. Pil proteins in this model are represented by their single letter designations. OM, outer membrane; IM, inner membrane. This integrated T4P structure, which consists of the indicated Pil protein at the minimum, may exist in the absence of the pilus filament. The interactions of PilB with PilC and PilM are inferred from genetic analysis and they may be either direct or indirect. See main text for details.

Our analysis of genetic suppression here suggested an integrated T4P structure in *M. xanthus* (Figure 6). A systematic Y2H analysis detected the following pairwise interactions among *M. xanthus* Pil proteins: PilQ-PilQ, PilQ-PilP, PilP-PilO and PilO-PilN. Since PilP is an IM lipoprotein and PilN as well as PilO are integral IM proteins, these interactions allow the OM protein PilQ to communicate with the IM T4P proteins. PilM, while cytoplasmic, is likely anchored to the IM by binding to the cytoplasmic tail of PilN [44], [56]. These results support a model of an integrated T4P structure in *M. xanthus* because the ability of PilB^WA^ to signal for EPS production requires all of these proteins. This integrated structure would include PilQ in the OM, the TM proteins PilN, PilO and PilC as well as the lipoprotein PilP on the IM. The cytoplasmic proteins PilM and PilB may associate with this structure dynamically as indicated by the genetic suppression patterns by *pilB^WA^*. The interactions among these proteins in *M. xanthus* apparently occur in the absence of the pilus filament as *pilB^WA^* suppresses Δ*pilA* but none of the other *pil* deletions.

Using the same Y2H system, we extended the above interactions to T4P proteins from the non-proteobacterium *T. thermophilus*. It is especially noteworthy that TtPilW shows interactions with the same T4P proteins as PilP even though they share no detectable structural similarity *in silico*. Interestingly, the more detailed interactions of TtPilW and TtPilQ resembles those of GspC and GspD in T2SS instead of *M. xanthus* PilP and PilQ. That is, both PilW and GspC interact with the N0 subdomains of their partner secretin. Similar observations were made recently between PilP and PilQ in *P. aeruginosa* and *N. meningitidis* [56], [57]. The findings with *T. thermophilus* T4P proteins suggest that the interactions among T4P proteins and the formation of an integrated T4P structure are conserved across different bacterial lineages despite extensive sequence and structural divergence. These observations suggest that a discovery in one bacterium may be applicable to related T4P systems in others even when they are evolutionarily distant. While the interactions reported here are mostly consistent with previous reports [[14], [56], [57] and references therein], they are by no means exhaustive as indicated by a recent report on *N. meningitidis* T4P protein-protein interactions using the bacterial two-hybrid (B2H) system in *E. coli* [17]. Substantially more interactions were detected in the B2H system, which may require additional verification by alternative approaches because *E. coli* shares many proteins with *N. meningitidis* than does yeast with bacteria.

The results here are supportive of other reports in the literature. A very recent publication based on *in vitro* experiments concluded that *P. aeruginosa* PilM, PilN, PilO, PilP and PilQ form a transenvelope network that interact with PilA [56]. The results here clearly indicate that a T4P complex can exist independently of PilA or the pilus filament. It was previously observed that certain T4P proteins localize to both cell poles in *M. xanthus* even though only one of the two poles may actively assemble and disassemble T4P at any given moment [40]. The existence of a T4P complex independent of the pilus filament provides support that the T4P proteins localized to the un-piliated pole may be organized into a complex standing ready for T4P assembly for the directional reversal of T4P mediate bacterial surface motility [38], [40].
