# Correction: Type IV Pilus Proteins Form an Integrated Structure Extending from the Cytoplasm to the Outer Membrane

**DOI:** 10.1371/journal.pone.0107344

**Published:** 2014-09-02

**Authors:** The PLOS ONE Staff

In the Funding section, the grant number from the funder National Science Foundation is missing. The correct grant number is: 1239889.
